# Psychometric Properties of Sinhala Version of Short-Form McGill Pain Questionnaire-2 (SF MPQ-2-Sin) among Patients with Cancer Pain in Sri Lanka

**DOI:** 10.1155/2019/5050979

**Published:** 2019-10-08

**Authors:** Nirosha P. Edirisinghe, Thamasi R. Makuloluwa, Thamara D. Amarasekara, Christine S. E. Goonewardena

**Affiliations:** ^1^Faculty of Graduates Studies, University of Sri Jayewardenepura, Nugegoda, Sri Lanka; ^2^Department of Clinical Sciences, Faculty of Medicine, General Sir John Kotelawala Defence University, Colombo, Sri Lanka; ^3^Department of Nursing and Midwifery, Faculty of Allied Health Sciences, University of Sri Jayewardenepura, Nugegoda, Sri Lanka; ^4^Department of Community Medicine, Faculty of Medical Sciences, University of Sri Jayewardenepura, Nugegoda, Sri Lanka

## Abstract

**Introduction:**

Pain is one of the most common and unpleasant symptoms that distress the well-being of patients with cancer. Considerable evidence supports the validity and reliability of the McGill Pain Questionnaire (MPQ) and its short forms, the SF MPQ and SF MPQ-2—which are the most widely used tools for pain assessment—in terms of patients with cancer. Pain and its characteristics are best assessed using validated and culturally adapted tools developed in participants' mother tongue. Although many pain assessment tools are available worldwide, only a limited number of them have been translated into Sinhala language and validated in Sri Lanka. We aimed to translate SF MPQ-2 into Sinhala language and validate using Sinhala-speaking patients suffering from cancer pains in Sri Lanka.

**Materials and Methods:**

Translation has been conducted according to the guidelines laid down by Mapi Research Trust, in five stages, namely, forward translation, backward translation, expert opinion, cognitive debriefing interviews, and proofreading. The questionnaire was administered among 207 patients attending Apeksha Hospital, Sri Lanka, who are suffering from cancer pain. Content validity was tested using expert opinion, and face validity, by interviewing patients with cancer pain. Factor structure was tested through a factor analysis, and reliability, by internal consistency with Cronbach's alpha.

**Results:**

A total of 207 participants (112 males and 95 females), aged between 20 and 80 years, were included in the study. Factor analysis identified four factors compatible with studies done in other countries, which explained 53.5% of the variance. The analysis of data indicated Cronbach's alpha of neuropathic, affective, intermittent, and continuous subscales as 0.768, 0.791, 0.824, and 0.789, respectively, which were over the acceptable threshold of 0.70. Confirmatory factor analysis supported the four-factor model.

**Conclusion:**

SF MPQ-2-Sinhala version is a statistically proven reliable and valid pain descriptor which can be utilized to evaluate pain suffered by patients with cancer in Sri Lanka whose mother tongue is Sinhala.

## 1. Introduction

Pain, which is one of the most common symptoms encountered by patients with cancer, is defined by the International Association for the Study of Pain [1] as an unpleasant sensory and emotional experience associated with actual or potential tissue damage or described in terms of such damage. Evaluation and assessment are the first steps of any strategy for the management of cancer pain and are the fundamentals of any clinical research project [2]. Visual analogue scales, verbal and numerical rating scales, and some multidimensional tools, such as the Brief Pain Inventory (BPI) and the McGill Pain Questionnaire (MPQ), are helpful in the assessment of different dimensions of cancer pain. Assessment tools are needed to be validated in the specific patient groups that are being studied [3].

McGill Pain Questionnaire (MPQ) long form was developed in 1975 by Melzack, enabling measuring sensory, affective, and evaluative qualities of pain [4, 5], which has demonstrated excellent reliability and validity in many types of acute and chronic pain conditions. MPQ comprising 78 pain descriptors is time consuming to be used, and therefore, authors developed the short-form MPQ (SF MPQ) in 1987 with 15 pain descriptors inclusive of visual analogue and verbal rating scales of pain intensity [6]. Dworkin et al., in 2009 expanded SF MPQ by adding seven more neuropathic pain descriptors, and the latest version, SF MPQ-2, includes 22 pain descriptors with four subscales: one affective and three sensory. Affective subscale includes four descriptors: tiring/exhausting, sickening, punishing-cruel, and fearful. The sensory subscales consist of three types of pains: continuous pain, intermittent pain, and neuropathic pain. Continuous pain includes throbbing pain, cramping pain, gnawing pain, aching pain, heavy pain, and tender. Intermittent pain includes shooting pain, stabbing pain, sharp pain, splitting pain, piercing pain, and electric shock pain. Neuropathic pain is categorized as hot burning pain, cold freezing pain, pain caused by light touch, itching, tingling of pins and needles, and numbness. All pain descriptors are rated on a 0 to 10 (none to worst possible pain) numerical rating scale according to the severity of pain perceived or felt. SF MPQ-2, translated and validated in many languages, shows well-established reliability and validity enabling its use in clinical and research settings [7]. Many studies conducted in the past have emphasized its reliability, validity, and responsiveness in patient samples of different pain conditions [8–13].

According to the Annual Health Statistics 2016, neoplasms are ranked as the second leading cause of deaths in Sri Lanka since 2009 [14]. Meegoda et al. conducted a descriptive cross-sectional study in Sri Lanka among 124 patients with cancer, and the majority (68%) reported pain relief as the most common reason for their readmissions [15]. Although several studies on pain related to cancers are being conducted in Sri Lanka, no multidimensional instrument in the Sinhala language—with proven reliability and validity—is currently available to assess pain. In view of cultural differences, translated pain scales must be appropriately validated to make them suitable for the corresponding culture [16]. In Sri Lanka, where the majority speaks Sinhala as their mother tongue, the availability of a culturally adapted and validated tool in the Sinhala language will facilitate the assessment of cancer pain by improving the quality of pain management. This study, therefore, intends to evaluate the reliability and validity of SF MPQ-2-Sinhala version as a pain descriptor to be used among Sinhala-speaking adults in Sri Lanka who suffer from cancer pain.

## 2. Materials and Methods

### 2.1. Study Population

Subjects were selected using the consecutive sampling method, from patients who suffer from cancer pain and who attend the Pain Clinic of Apeksha Hospital (National Cancer Institute), Maharagama, the premier cancer treatment centre in Sri Lanka. Patients over 18 years of age, who were diagnosed with any type of cancer and who are suffering from pain related to primary lesion, secondaries, radiotherapy, or chemotherapy for at least a duration of 3 months, were included in the study. Out of these patients, the patients of different ethnic groups and who are capable of comprehending Sinhala language were chosen. Patients, whose pain is a result of any cause other than cancer triggered within a month from the time of assessment and, those who were frail, mentally unfit, disoriented with evidence of brain metastases and are unable or unwilling to give informed consent were excluded from the study. The sample size was calculated considering the rule of thumb of 5–10 subjects per item [17]. There were 22 pain descriptors in SF MPQ-2-Sinhala version and the calculated sample size for the validation study was 220 (Sample 1). Data were collected from another set of 384 subjects, for confirmatory factor analysis (CFA), from the same study setting, adhering to the same inclusion-exclusion criteria used to recruit participants (Sample 2). Those who were included in the sample 1 were excluded from sample 2, in order to prevent contamination.

### 2.2. Translation of SF MPQ-2 English Version to Sinhala Version

The translation was carried out in 5 steps: forward translation, back translation, review by experts, cognitive debriefing, and proofreading as per guidelines given by original authors of Mapi Research Trust. The forward translation was conducted by two native translators fluent in both languages. Out of them, a language expert and a subject expert were selected in order to produce the best possible translated version of the questionnaire. Two translators were not known to each other, and translations were done individually and separately. The two forward Sinhala translations were compared with each other and with the original English version by the PI and the experts who were fluent in both languages. Backward translation was conducted by two independent translators who were blind to the original English version, and retranslated the Sinhala into English language. One of them was a professional translator and the other was a consultant anaesthetist specialized in pain medicine. Content validity of the Sinhala version was carried out using the Modified Delphi Technique [18]. Face validity of the SF MPQ-2-Sinhala version was assessed by conducting cognitive debriefing interviews with patients with cancer pain. As the final step of the process, proofreading was conducted to correct grammar, spelling, typographic, and formatting errors.

### 2.3. Method of Data Collection

The principal investigator collected data from September 2017 to June 2018, using the translated SF MPQ-2-Sinhala version as an interviewer-administered questionnaire. Data on demographic characteristics, cancer type, treatments, duration of pain, duration of disease, and other comorbidities like diabetes and hypertension were also collected using an interviewer-administered questionnaire.

### 2.4. Statistical Analysis

Statistical analysis was performed using SPSS version 20.0 for windows. Demographic data were analyzed by means of descriptive statistics. For all tests, *p* < 0.05 was considered statistically significant. Factor structure was tested by exploratory factor analysis (EFA) with direct oblimin rotation, and a value of 0.4 was considered mandatory for the loading of each factor, considering the sample size [19]. The Kaiser–Meyer–Olkin (KMO) measure and Bartlett test of sphericity were analyzed to appraise the appropriateness of data for factor analysis, and KMO values of ≥0.70 were considered average [20]. Reliability was tested with internal consistency, using Cronbach's alpha, and coefficients of ≥0.70 were considered to possess an acceptable internal consistency [21].

#### 2.4.1. Confirmatory Factor Analysis

CFA was performed using Lisrel 10.20 for windows to evaluate the adequacy of the models. Four-factor model was evaluated, and the indices used to evaluate model fit include the absolute fit indices (Satorra–Bentler scaled chi-square test, root mean square error of approximation (RMSEA), goodness of fit index (GFI), adjusted goodness of fit index (AGFI), and standardized root mean square residual (SRMR)), relative fit indices (comparative fit index (CFI) and nonnormed fit index (NNFI)), and parsimony fit indices (parsimony goodness of fit index (PGFI) and parsimonious normed fit index (PNFI)). RMSEA values of <0.05 indicate a good fit to the data; values between 0.05 and 0.08 are an acceptable fit; values between 0.08 and 0.10, a marginal fit; and values >0.10, a poor fit [22]. For the CFI and NNFI, values >0.95 indicate a good fit to the data while for GFI and AGFI are >0.90 [23]. For PGFI and PNFI, values >0.5/no absolute threshold values were considered [24].

### 2.5. Ethical Considerations

Approval was obtained for the translation of SF MPQ-2 from Mapi Research Trust, France, and ethical approval was obtained from the Ethics Review Committee, Faculty of Medical Sciences, University of Sri Jayewardenepura, Sri Lanka. Permission for data collection was obtained from the Director, Apeksha Hospital, Maharagama, and informed written consent was obtained from all the study participants.

## 3. Results

### 3.1. Sample Characteristics

As shown in Table 1, the study population consists of 207 participants in sample 1, 54.1% (*n* = 112) males and 45.9% (*n* = 95) females in the age range of 18–80 years with an average age of 54.2 years (SD ±13.2). Thirteen participants' data were incomplete for one or more items and, therefore, were excluded from the analysis. Sample 2 consists of 384 participants.

### 3.2. Reliability Analysis

Cronbach's alpha values for subscales of continuous, intermittent, neuropathic, and affective were 0.789, 0.824, 0.768, and 0.791, respectively. All the values for “alpha if item deleted” were lower than corresponding subscale values except for “Tiring-exhausting,” which showed a very small increase in alpha of 0.001. Therefore, the Cronbach's alpha coefficient revealed an acceptable internal consistency for the subscales of the SF MPQ-2-Sinhala version.

### 3.3. Factor Structure

The EFA indicated that the KMO value of the SF MPQ-2-Sinhala version to be 0.743, and factors extracted with eigenvalues of >1. Five factors with a variance of 58.4% were extracted, and only one item was loaded (pain to light touch, 0.639; eigenvalue, 1.057) for the 5^th^ factor. The scree plot revealed a clear break after the 4^th^ component. Using Cattell's scree test, it was decided to retain four components for further investigation [25]. This was further supported by the results of parallel analysis (Monte Carlo PCA) which showed only four components with eigenvalues exceeding the corresponding criterion values for a randomly generated data matrix of the same size [26]. Then factor analysis was conducted using the extraction methods with “fixed number of factors equal to 4.” Four factors with the variance explained 53.5% were extracted. The item “pain to light touch” was loaded into neuropathic pain subscale with the loading of 0.500 that was comparable with the original SF MPQ-2 subscales of continuous, intermittent, neuropathic, and affective (Table 2).

For CFA, the robust maximum likelihood method (RML), which was adjusted for nonnormality of the data, was used to estimate the model parameters. The four-factor model gave a chi-squared value of 410.971 (d*f* = 203) (*p* < 0.000), and the absolute fit indices were as follows: RMSEA = 0.0517, GFI = 0.916, AGFI = 0.895, and SRMR = 0.0606. Relative and parsimony fit indices were as follows: CFI = 0.829, NNFI = 0.806, PGFI = 0.735, and PNFI = 0.630. The four-factor model came up with better indices closer to the desired values in absolute, relative, and parsimony fit indices.

## 4. Discussion

The SF MPQ-2 is one of the most widely used multidimensional pain scales, which is an extension of the SF MPQ, which contains the most common descriptions of neuropathic pain experienced by patients. This study set out to evaluate the psychometric properties of the Sinhala version of SF MPQ-2 as a pain descriptor among Sinhala-speaking patients in Sri Lanka who suffer from cancer pain. Five steps, i.e., forward translation, back translation, review by experts, cognitive debriefing, and proofreading, were followed in the translation of SF MPQ-2 to obtain an acceptable face and content validity. Content validity was tested by the Modified Delphi Technique. Experts comprised nurses and consultants who are involved in pain management in Sri Lanka, and each item of the questionnaire was assessed on relevance, appropriateness, and acceptability. Three iterative cycles were conducted in order to reach the agreement. Face validity of the SF MPQ-2-Sinhala version was assessed by conducting cognitive debriefing interviews using a five-point Likert scale with regard to clarity and fluency of each item, where 0 is the least and 4 is the maximum score. A mean of ≥3 for each item was indicative of clarity and fluency. Cognitive debriefing interviews were conducted with ten patients diagnosed with different types of cancer pain, who fall under different age groups and various educational levels, domiciled in different provinces of the country. The interviews were directed to each item in the questionnaire separately, in order to determine whether the word flow of the questions that had been used has made any of the items difficult to understand, confusing, difficult to answer, objectionable, and, according to them, if the question could have been asked in a different way to improve comprehensibility. Based on the comments of the respondents, minor changes were made to the document without affecting the meaning of the items. Face validity testing needed no further major revisions regarding the item content or scoring. Apeksha Hospital Maharagama, the premier treatment centre for cancer patients in Sri Lanka, was chosen as the study setting for sampling, due to the ethnic-geographic and cultural diversity of its patients. The results of this study can be generalized to larger groups of patients undergoing cancer pain, as the patients included in this study, who were suffering from pain, were patients with different cancer diagnoses.

Reliability analysis was conducted by testing the internal consistency using Cronbach's alpha. Cronbach's alpha coefficients were computed for each subscale and ranged from 0.76 to 0.82; all of which lie above the commonly accepted thresholds for internal consistency. The results are comparable with those of the original study and other language versions, as illustrated in Table 3 [7, 9, 10, 12, 27]. Majority of the items appeared to be worthy of retention, resulting in a decrease in “alpha,” if deleted. One exception to this was the item “tiring/exhausting,” where the alpha would increase (*α* = 0.792) by a value of 0.001 if deleted. However, as the item “tiring/exhausting” is a frequently used term among patients with pain, having considered this fact, it was decided to retain the item “tiring/exhausting” in the questionnaire.

EFA was carried out to explore the possible underlying factor, and CFA was used to verify the factor structure. Identification of factor structure was conducted with the sample 1 data, and verification of factor, with sample 2 data, considering the recommendations [28, 29]. Factor analyses of the SF MPQ-2 in several other languages have identified continuous pain, intermittent pain, affective pain, and neuropathic pain items loading on the four factors [8–13]. Initially, five factors which loaded only one item to the fifth factor were found with eigenvalue just above 1 (1.057). Following the parallel analysis performed, only four components demonstrated eigenvalues exceeding the corresponding criterion values. Therefore, we have chosen the extraction method of “fixed number of factors,” which produced four-factor solution, consistent with the original structure and validation of the SF MPQ-2 [7–13]. The correlation coefficient score for some items in EFA was lower than that for the others (Table 2), which may be result of an insignificant unclearness of some words specific to the Sinhala language/culture. Conversely, some words or expressions may be intrinsically challenging to comprehend. According to the CFA results, an acceptable model fit was observed, and the values of the fit indices for four-factor model were acceptable and closer to the desired level. Therefore, it is safe to conclude that four-factor model best fits and explains the items of the SF MPQ-2-Sinhala version in the tested sample.

One of the limitations of the study is that the patients who participated in it were being diagnosed with different types of cancers, with reported pains in different sites of their body. In order to widen generalizability of results, further studies are recommended to be conducted among the patient groups with different types of cancers with reported pain in different sites.

## 5. Conclusion

In conclusion, SF MPQ-2-Sinhala version is a statistically proven, reliable, and valid pain descriptor, which can be used in clinical research as well as in the routine clinical evaluation of Sinhala-speaking patients presenting with pain related to cancers.

## Figures and Tables

**Table 1:** Sample characteristics.

Demographic characteristics		Sample 1 (*n* = 207)		Sample 2 (*n* = 384)	
		Frequency	Percentage	Frequency	Percentage
Age		Mean = 54.24, SD = ±13.18, range = 20–80 years		Mean = 56.17, SD = ±11.83, range = 19–88 years	

Gender	Male	112	54.1	153	39.8
	Female	95	45.9	231	60.2

Race	Sinhala	182	87.9	333	86.7
	Tamil	16	7.7	33	8.6
	Muslim	4	1.9	15	3.9
	Burger	4	1.9	3	0.8
	Malay	1	0.5	—	—

Level of education	Not attended school	11	7.2	22	5.7
	Up to grade 5	85	41.1	150	39.1
	Up to ordinary level	71	34.3	127	33.1
	Up to advanced level	42	20.3	76	19.8
	Graduate	5	2.4	6	1.6
	Postgraduate	4	1.9	3	0.8

Marital status	Single	17	8.2	27	7.0
	Married	158	76.3	306	79.7
	Divorced	4	1.9	14	3.6
	Living together	1	0.5	—	—
	Widow	27	13.0	37	9.6

**Table 2:** Factor structures and loadings of 22 items in SF MPQ-2.

Item	Extraction method: fixed number of factors			
	Factor 1	Factor 2	Factor 3	Factor 4
Throbbing pain	−0.758	0.065	0.067	0.210
Gnawing pain	−0.567	0.068	−0.063	−0.087
Aching pain	−0.722	−0.025	0.003	−0.147
Cramping pain	−0.568	−0.274	−0.023	−0.038
Heavy pain	−0.782	−0.072	−0.043	0.009
Tender	−0.743	0.082	0.061	−0.035
Shooting pain	−0.036	0.720	−0.019	−0.024
Stabbing pain	0.064	0.738	0.108	0.021
Sharp pain	0.025	0.785	0.055	−0.142
Piercing	0.046	0.712	−0.030	0.010
Electric shock pain	−0.047	0.706	−0.140	0.078
Splitting pain	−0.035	0.713	0.031	0.033
Tiring, exhausting	−0.038	0.048	0.703	−0.053
Sickening	−0.059	−0.024	0.780	−0.063
Fearful	0.051	−0.046	0.843	0.062
Punishing, cruel	0.038	0.009	0.814	0.075
Cold freezing	−0.047	0.021	0.046	0.700
Numbness	−0.127	−0.031	−0.065	0.809
Hot burning pain	0.064	−0.029	0.089	0.673
Itching	0.077	−0.130	0.010	0.669
Tingling or “pins and needles”	0.065	−0.014	−0.045	0.693
Pain caused by light touch	0.027	0.167	−0.025	0.500
Eigen values	3.921	3.094	2.493	2.271
% of variance	17.824	14.065	11.330	10.324
Total %	53.54%			

**Table 3:** Internal consistencies for each subscale in the current study, original study, and other studies.

Study	Continuous	Intermittent	Affective	Neuropathic
Current study	0.79	0.82	0.79	0.77
Original study	0.87	0.87	0.86	0.83
Thai version	0.79	0.80	0.89	0.77
Acute low back pain	0.77	0.82	0.84	0.80
Persian version	0.75	0.81	0.81	0.58
Japanese version	0.89	0.87	0.85	0.91

## Data Availability

The data used to support the findings of this study are available with the corresponding author upon request.
